# Effects of Stress Level and Elevated Temperature on Transverse Compression Stress Relaxation Behavior and Post-Relaxation Mechanical Performance of UD-CFRP

**DOI:** 10.3390/polym17202718

**Published:** 2025-10-10

**Authors:** Jianwen Li, Maoqiang Wang, Lili Hu, Xiaogang Liu

**Affiliations:** 1 Research Institute of Urbanization and Urban Safety, School of Future Cities, University of Science and Technology Beijing, Beijing 100083, China; d202110028@xs.ustb.edu.cn (J.L.); hll_1010@163.com (L.H.); 2 Highway Bridges National Engineering Research Center, Beijing 100120, China; wangmaoqiang@bnerc.com

**Keywords:** UD-CFRP, transverse compression, stress relaxation, post-relaxation property

## Abstract

Unidirectional carbon fiber-reinforced polymer (UD-CFRP) composites demonstrate superior tensile creep strain and stress relaxation behavior along fiber orientation. However, prolonged transverse compressive loading in structural connection zones induces significant interfacial stress relaxation and creep deformation, primarily driven by resin matrix degradation and interfacial slippage under thermal-mechanical interactions, and remains poorly understood. This study systematically investigates the transverse stress relaxation characteristics of UD-CFRP through controlled experiments under varying thermal conditions (20–80 °C) and compressive stress levels (30–80% ultimate strength). Post-relaxation mechanical properties were quantitatively evaluated, followed by the development of a temperature-stress-time-dependent predictive model aligned with industry standards. The experimental results reveal bi-stage relaxation behavior under elevated temperatures and compressive stresses, characterized by a rapid primary phase and stabilized secondary phase progression. Notably, residual transverse compressive strength remained almost unchanged, while post-relaxation elastic modulus increased by around 10% compared to baseline specimens. Predictive modeling indicates that million-hour relaxation rates escalate with temperature elevation, reaching 51% at 60 °C/60% stress level—about 1.8 times higher than equivalent 20 °C conditions. These findings provide crucial design insights and predictive tools for ensuring the long-term integrity of CFRP-based structures subjected to transverse compression in various thermal environments.

## 1. Introduction

Steel cables, while widely used in structural applications, are inherently limited by their substantial mass, corrosion vulnerability, and fatigue degradation. Unidirectional carbon fiber-reinforced polymer (UD-CFRP) composites present a viable alternative, offering superior strength-to-weight ratio, corrosion resistance, and fatigue performance [1,2]. In prestressed cable applications, UD-CFRP effectively utilizes its anisotropic characteristics to achieve optimal tensile strength along the fiber direction [3,4]. The material demonstrates exceptional fatigue resistance under cyclic loading [5,6] and corrosion stability in aggressive environments [7,8], with impact performance enhanced through protective sheathing [9,10]. These attributes have led to successful implementations in bridge engineering, tensioned roof structures, and structural reinforcement systems [11,12].

UD-CFRP composites, comprising aligned fibers embedded in a thermosetting resin matrix, demonstrate intrinsic time-dependent viscoelastic behavior manifested as creep strain and stress relaxation [13]. While fiber-dominant tensile creep along the longitudinal axis remains negligible [14], effective anchorage systems prove critical for tension capacity development. Persistent transverse compressive stresses from clamping mechanisms activate resin-dominated viscoelastic responses, resulting in sustained relaxation [15,16]. This stress decay mechanism directly correlates with the anchor system-CFRP interfacial anchoring stresses, potentially diminishing anchor system performance through progressive shear slip accumulation [15].

Recent investigations into UD-CFRP’s viscoelastic responses under sustained loading have established fundamental mechanisms governing tensile stress relaxation and creep development [13,17]. During longitudinal tensile creep, primary creep derives from resin matrix viscoelasticity and wrinkled fiber straightening, while interface shear creep dominates secondary stages, which also mainly arises from resin matrix. Critical stress thresholds (around 70–85% ultimate strength) precipitate fiber fracturing through cumulative damage, ultimately causing creep rupture. Established relationships demonstrate creep strain rate’s linear proportionality to applied stress magnitude [13,14] and logarithmic time-dependence [18,19]. The anisotropy of UD-CFRP dictates a contrasting creep mechanism of fiber-governed behavior under axial tension [20].

However, under transverse compression, the creep response becomes resin matrix-controlled and more pronounced [15]. In cable anchorage systems, UD-CFRP is subjected to sustained transverse compressive stresses from mechanical clamps, generating resin-dominated transverse creep, thus progressively inducing mechanical clamping stress relaxation and triggering anchorage slippage. Consequently, the anchorage slippage causes tensile prestress loss in UD-CFRP through gradual stress redistribution.

Experimental characterization of UD-CFRP’s transverse compressive stress relaxation at ambient conditions demonstrates biphasic decay patterns across critical stress thresholds (30–80% ultimate strength) [21]. Initial rapid relaxation (within around 24 h) stems from resin matrix void closure, followed by progressive time-dependent relaxation governed by viscoelastic creep of the resin matrix and fiber-matrix interfaces. Comparatively, biaxial fabric composites exhibit initial creep transients (in 12–24 h) followed by secondary creep phases at a diminished rate over 150 h under equivalent loading [22]. Multi-scale modeling frameworks confirm matrix-dominated creep mechanics in GFRP laminates under transverse compression, revealing significant stress-amplified creep compliance through resin phase nonlinear viscoelasticity [23].

Under thermal exposure, the resin matrix exhibits enhanced viscoelasticity [24], significantly influencing the creep and stress relaxation behavior of FRP composites. However, research on UD-FRP under coupled thermo-mechanical conditions remains limited. Experimental studies on UD-CFRP tendons under 25–300 °C and 20–60% stress levels demonstrate temperature-proportional creep and stress relaxation rates during the initial 4 h stage, followed by temperature-insensitive rates in subsequent stable stages [25,26]. Meanwhile, post-creep residual tensile strength shows dual dependence, decreasing with elevated sustained stress levels and temperatures. Comparative tests reveal that biaxial fabric composites under 80 °C transverse compression exhibit significantly greater creep deformation than those at ambient temperatures [22].

Current investigations on transverse compressive creep and stress relaxation of UD-CFRP under elevated temperatures remain insufficient, particularly concerning coupled thermo-mechanical conditions. Previous studies have established that elevated temperatures and increased sustained stress levels significantly amplify these time-dependent behaviors in UD-CFRP [21,22,23]. In practical cable anchorage systems, UD-CFRP experiences persistent transverse clamping stress during its service life while simultaneously enduring environmental thermal loads (e.g., solar radiation). This necessitates a comprehensive investigation into the long-term transverse stress relaxation behavior of UD-CFRP under combined thermal-mechanical conditions to establish evaluation criteria for anchorage system degradation. Adhering to BS ISO 20975-1 [27], this study implements 1000 h transverse compressive stress relaxation tests under varying temperatures and stress levels, followed by post-test static compressive property characterization. A predictive model incorporating temperature, stress level, and duration effects is developed to forecast million-scale service hour relaxation behavior. This study provides much-needed data and a practical predictive framework for the design and integrity assessment of CFRP-based anchorage systems under real-world service conditions.

## 2. Experimental Program

### 2.1. Materials

**Figure 1:**
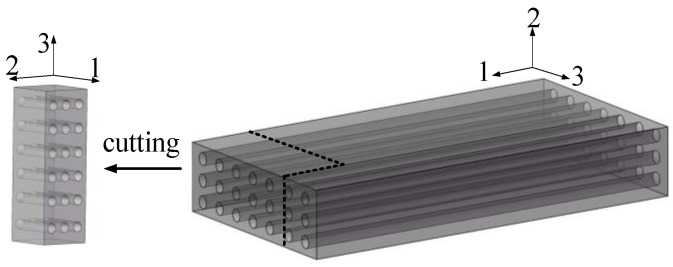
Preparation of test specimens.

The experimental UD-CFRP composites (The materials were sourced from Zhongfu Carbon Core Cable Technology Co., Ltd. (Lianyungang, China)) were manufactured through a pultrusion process with cross-sectional dimensions of 16 mm × 60 mm and a fiber volume fraction of 72%. As detailed in Table 1, the constituent materials (carbon fibers and epoxy resin) possess distinct mechanical properties. Specimens were precision-machined (The precision machining equipment (Model: JK-DK40) was sourced from Changzhou Jinghua Numerical Control Equipment Co., Ltd., Changzhou, China) from CFRP profiles (Figure 1) to dimensions of 16 mm (1st fiber direction), 16 mm (2nd in-plane transverse direction), and 40 mm (3rd through-thickness direction) [27], aligning with principal material axes for third-direction (through-thickness) sustained loading. Specimen preparation involved diamond-tool sectioning using milling equipment under controlled feed rates (<0.1 mm/s) to minimize machining-induced defects. Post-mortem SEM characterization revealed preserved fiber continuity within gauge regions, validating specimen integrity. The inter-specimen consistency in relaxation rate evolution further confirms the reliability of the fabrication protocol.

### 2.2. Experimental Set-Up

**Figure 2:**
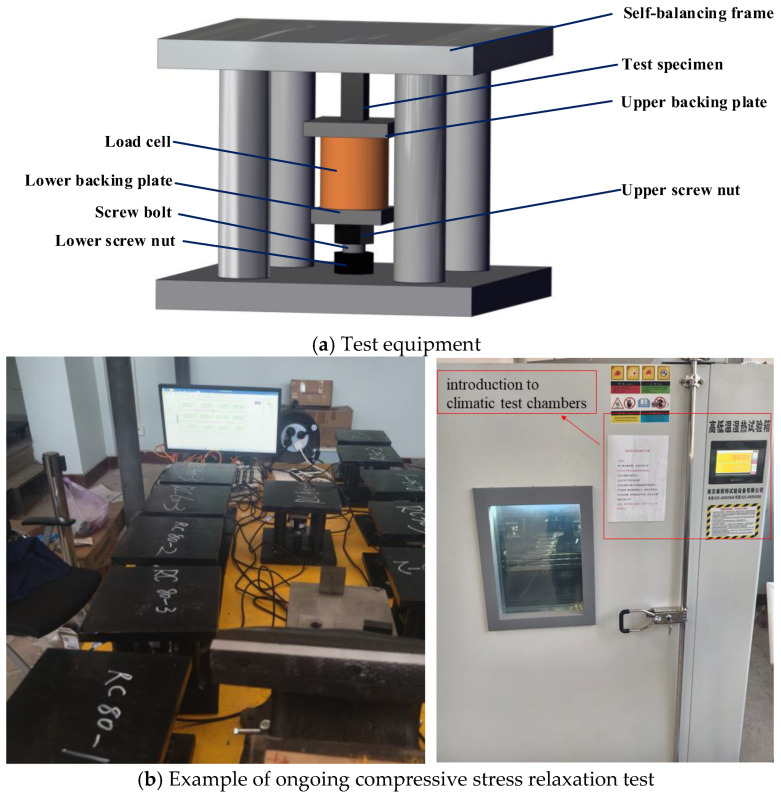
Test set-up and specimens.

The transverse compressive stress relaxation testing of UD-CFRP specimens under specified initial stress requires maintaining constant compressive strain while monitoring stress variations. A custom testing apparatus (Figure 2) was developed for this purpose, comprising a self-balancing frame, screw bolt, nut assemblies, and a through-hole load cell. The frame’s base plate contains a central threaded hole to secure the screw bolt via a lower nut, while an upper nut adjusts vertically to apply initial compressive load. The load cell, positioned between the specimen and upper nut, continuously records stress relaxation data.

To ensure testing accuracy [28], the frame, backing plates, and screw bolts were designed with high stiffness to minimize system deformation, thereby maintaining fixed compressive strain in the specimens. Testing was conducted in a temperature-controlled furnace following this procedure: (1) stabilize the furnace (The climatic test chamber was sourced from Nanjing Test Test Equipment Co., Ltd. (Nanjing, China), with the model number GDS-015) at the target temperature; (2) apply predefined transverse compressive stress using the apparatus; and (3) place the apparatus into the furnace and monitor stress relaxation over 1000 h under constant thermal conditions.

### 2.3. Specimens

Test specimens were fabricated in accordance with JSCE-534 [29] and ASTM D2990 [30], employing four thermal conditions (20 °C, 40 °C, 60 °C, 80 °C) and four initial transverse compressive stress levels (30%, 40%, 60%, 80% of UD-CFRP’s static compressive strength: 157 MPa). Each testing configuration consisted of 3 replicates, yielding 48 specimens in total, with specimen IDs and corresponding stress-temperature combinations detailed in Table 2. All specimens underwent 1000 h stress relaxation testing under controlled thermal exposure. Subsequent to relaxation testing, residual transverse compressive strength and elastic modulus were determined through static compression testing of post-relaxation specimens. Furthermore, SEM analysis was conducted on the fracture surfaces of the specimens after static compressive failure. These SEM results were compared with those obtained from the fracture surfaces of pristine specimens after static failure for detailed analysis.

## 3. Test Results and Discussions

### 3.1. Relaxation Process

**Figure 3:**
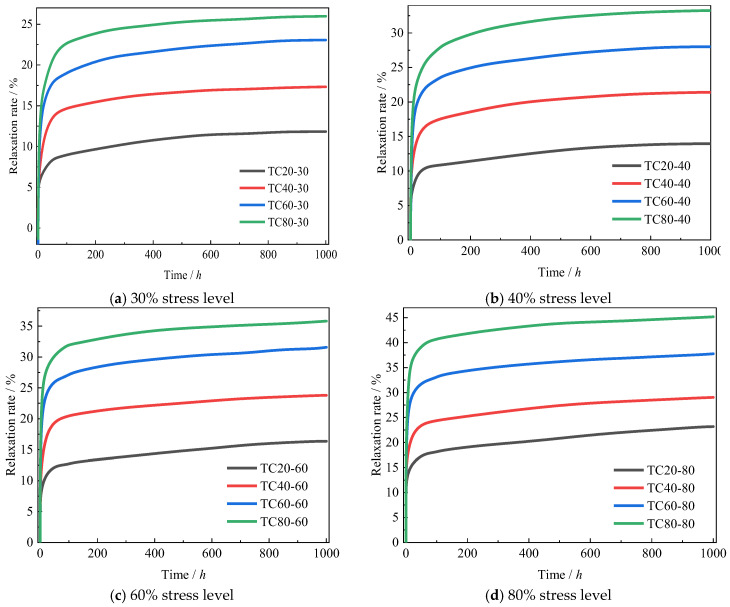
Stress relaxation under different temperatures and stress levels.

Figure 3 presents the temporal evolution of transverse compressive stress relaxation rates under varying temperature-stress combinations. All specimens exhibited a biphasic relaxation pattern during the 1000 h testing period: an initial rapid development stage followed by a stabilized progression. The primary stage demonstrates accelerated relaxation kinetics driven by thermo-mechanical coupling. This phase involves two concurrent mechanisms: (1) the compaction of UD-CFRP’s surface asperities, and (2) the viscoelastic collapse of resin matrix porosity. Localized stress concentrations at matrix defects (e.g., microvoids) induce plastic deformation and rapid creep of the resin matrix, subsequently redistributing internal stresses toward equilibrium. The resultant stress homogenization reduces defect-related stress concentrations, causing progressive deceleration of relaxation rates until reaching secondary stabilization. The duration of the primary stage shows temperature-stress dependency, with both elevated temperatures and higher stress levels prolonging this phase and intensifying relaxation rates. A quantitative analysis of these parametric relationships is provided in Section 3.2.

The secondary phase represents a quasi-stable evolution characterized by progressively decelerating relaxation rates. Post-1000 h testing revealed complete stabilization for 30% and 40% stress level specimens across all temperatures (20–80 °C), whereas 60% and 80% stress level groups maintained slight residual relaxation gradients. This stage’s relaxation mechanics originate from matrix-dominant viscoelastic flow in interfacial transition zones. Process-inherent defects (fiber waviness, resin-rich regions) further modify stress redistribution dynamics, affecting the relaxation process. Notably, thermal activation effects induced two observable phenomena: extended stabilization duration and amplified cumulative relaxation.

### 3.2. Relaxation Rate

**Figure 4:**
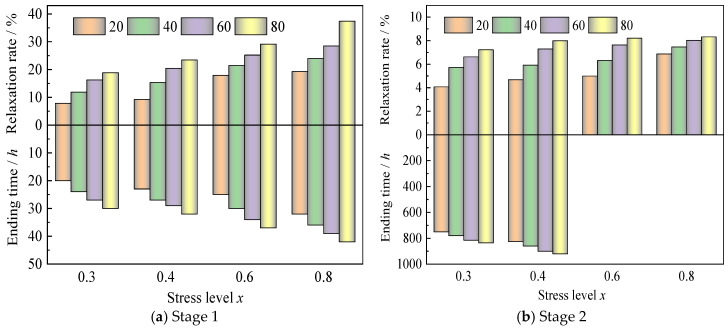
The ending time and relaxation rate under different temperatures and stress levels.

The relaxation rate, defined as the dimensionless characterization of stress decay (1 − *σ*_t_/*σ*_0_), demonstrates explicit temperature-stress dependencies as quantified in Figure 4 and Table 3. Two fundamental mechanisms govern these dependencies: (1) Thermal activation dominance: Elevated temperatures (40–80 °C vs. 20 °C) enhance molecular mobility [22], prolonging stabilization duration and amplifying relaxation magnitude at equivalent stress levels. (2) Stress level modulation: Higher initial stress (40–80% vs. 30%) also prolongs stabilization duration and amplifies relaxation magnitude at equivalent stress levels.

**Figure 5:**
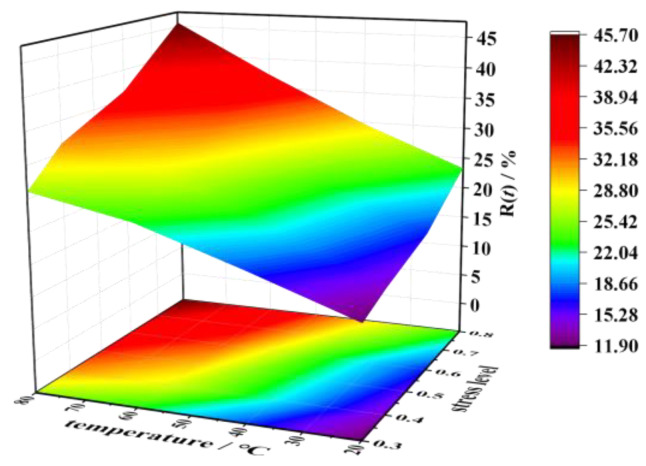
Trend of relaxation rate variation at different temperatures and stress levels.

**Figure 6:**
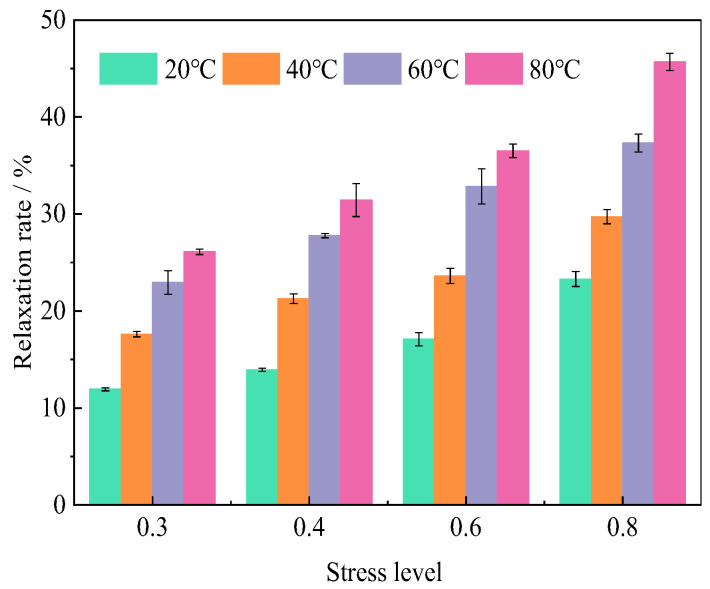
Comparison of relaxation rate under different temperatures and stress levels.

Figure 5 demonstrates the synergistic enhancement of stress relaxation rates with increasing both stress levels (30–80% ultimate strength) and temperatures (20–80 °C). This coupled thermo-mechanical behavior is quantitatively corroborated by 1000 h transverse compressive relaxation tests (Table 4 and Figure 6), revealing two systematic dependencies: (1) at fixed temperatures of 20 °C, 40 °C, 60 °C, and 80 °C, relaxation rates escalate by 2.01%, 3.66%, 4.83%, and 5.32% with stress level increments from 30% to 40%; (2) at an equivalent stress level of 60%, each 20 °C temperature elevation amplifies relaxation growth by 6.53%, 9.23%, and 3.66%. Notably, temperature dominates the relaxation rate over stress level contributions, especially beyond 60 °C, inducing greater relaxation enhancement than equivalent stress level increases (Figure 5).

### 3.3. Post-Relaxation Strength and Modulus

Post-relaxation static compressive testing (Table 5) revealed that while the original transverse compressive strength (157 Mpa) remained essentially unaffected by 1000 h relaxation under various temperatures (20–80 °C) and stress levels (30–80% ultimate strength), significant modulus evolution occurred. The post-relaxation modulus retention ratio *η* (a ratio of the post-relaxation modulus to the initial modulus) exhibited systematic enhancement: 10% increase under 30% stress level at 40–80 °C, 15% augmentation for 40–80% stress levels at 40–80 °C, and 10% elevation under all stress levels at 20 °C. Notably, specimens subjected to elevated temperatures (40–80 °C) with higher stress levels (40–80%) demonstrated around 4% greater modulus enhancement compared to their room temperature counterparts, whereas at a 30% stress level, the temperature-induced modulus gain reduced to 2%.

This phenomenon reveals a temperature-dominated but stress-assisted coupling effect on post-relaxation modulus enhancement. At room temperature (20 °C), the observed modulus improvement (around 10%) primarily arises from the stress-induced compaction of pore defects in the resin matrix [21]. Under elevated temperatures (40–80 °C), two concurrent mechanisms contribute to greater modulus enhancement: (1) thermal softening of the resin matrix accelerates poral defect closure (primary mechanism), and (2) limited secondary curing (resin’s glass transition temperature *T_g_* of 235 °C; Table 1) slightly increases crosslinking density [31]. Experimental evidence confirms that the thermal softening effect predominates, as temperatures within 80 °C induce minimal secondary curing. Crucially, compressive stress generates necessary stress concentration around poral defects to facilitate their permanent compaction. These coupled mechanisms explain the differential modulus enhancements: (1) around 14% improvement at 40–80% stress levels (40–80 °C) versus around 10% at a 30% stress level (40–80 °C); (2) both surpass room temperature gains by 2–4%.

**Figure 7:**
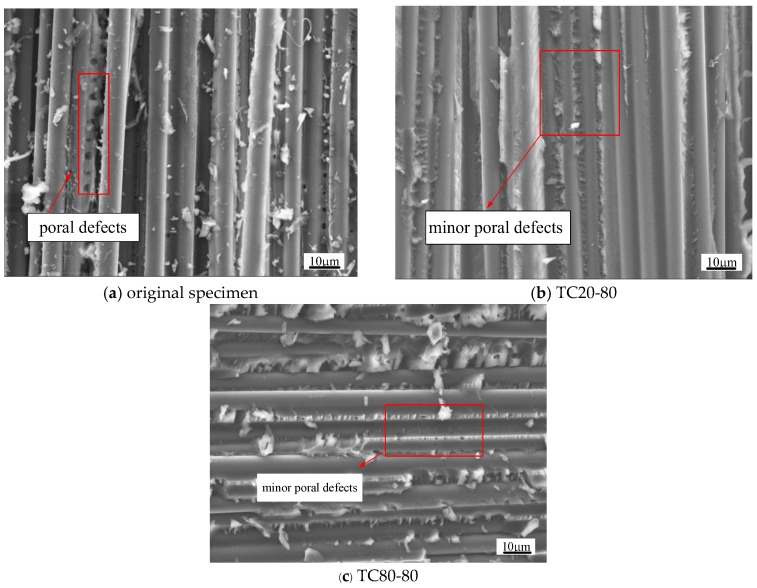
Comparative SEM images for different specimens after compression failure.

The fracture surface morphology In Figure 7a–c provides microscopic validation of these mechanisms. Comparative SEM observations (The scanning electron microscopy (SEM) observations were conducted using a JEOL JCM-7000 benchtop microscope (JEOL Ltd., Tokyo, Japan).) reveal distinct microstructural evolution: original specimens (Figure 7a) display clear resin matrix porosity, while post-relaxation counterparts (Figure 7b: 20 °C/80% stress level; Figure 7c: 80 °C/80% stress level) exhibit significantly reduced pore visibility, confirming stress- and temperature-enhanced compaction. Notably, tomentum-like failure patterns in post-relaxation specimens (Figure 7b,c) suggest intensified resin matrix deformation from creep processes and poral defect closure. Crucially, consistent fiber–resin interfacial morphology across all specimens (Figure 7a–c) corroborates preserved interfacial integrity, aligning with the observed stability in post-relaxation compressive strength.

## 4. Stress Relaxation Prediction

Experimental results demonstrate significant temperature-, stress level-, and time-dependent characteristics in stress relaxation development. In accordance with the JSCE-534 specification recommendation [29], the relaxation behavior can be effectively modeled using the logarithmic formulation presented in Equation (1).

(1) R(t)=a+blnt

where *R*(*t*) is the relaxation rate at a specific time *t*, with uint in %; parameter *a* means the relaxation value at 1 h, parameter *b* means a relaxation speed, and both parameters *a* and *b* are related to temperature level *ω* (*T*/*T_g_*) and stress level *x*; *t* represents time, with unit in hour.

As illustrated in Figure 5, the temperature and stress level effects exhibit no significant interdependence. Following the coefficient decoupling methodology prescribed in GB 51160-2016 [32], parameters *a* and *b* in Equation (1) are decomposed into distinct thermal (*a*(*ω*), *b*(*ω*)) and mechanical (*a*(*x*), *b*(*x*)) components. This analytical approach yields the modified formulation in Equation (2), where *ω* = *T*/*T_g_* represents the normalized temperature level, and *x* denotes the dimensionless stress level.

(2) R(t)=a(ω)a(x)+b(ω)b(x)lnt

Under isothermal conditions at 20 °C, the temperature-dependent coefficients *a*(*ω*) and *b*(*ω*) become united through normalization. This simplification transforms Equation (2) into the reduced-form Equation (3), which specifically characterizes the stress relaxation rate evolution under varying applied stress levels *x*.

(3) R(t)=a(x)+b(x)lnt

**Figure 8:**
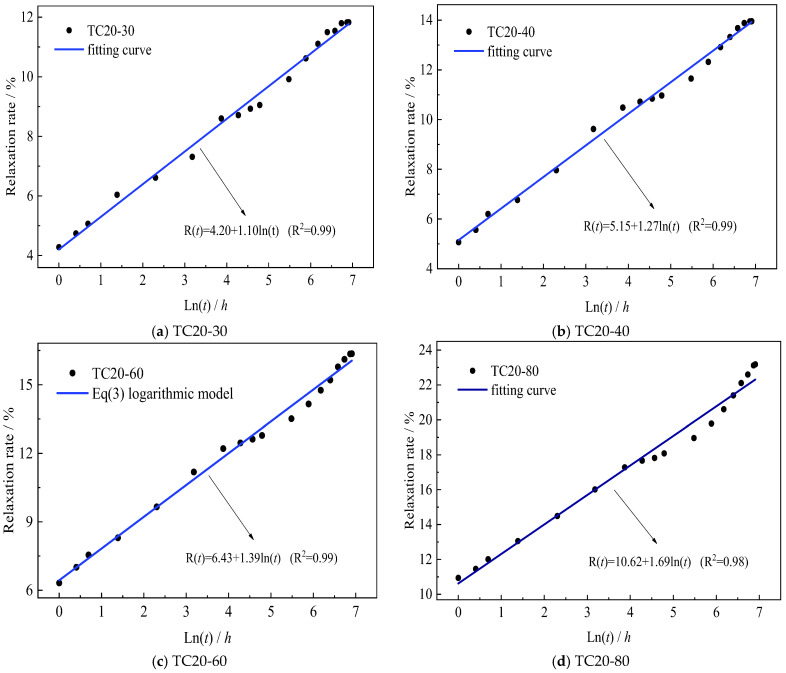
Relationship between *R*(*t*) and ln(*t*) under various stress levels.

The stress-dependent coefficients *a*(*x*) and *b*(*x*) were determined experimentally by fitting Equation (3) to isothermal test data obtained at 20 °C. The resulting values are presented in Figure 8 and Table 6. For TC20-series specimens with incremental stress levels (30%, 40%, 60%, 80%), the time-dependent relaxation rate *R*(*t*) demonstrates a strong linear correlation with ln(*t*), as statistically verified in Figure 8. The derived mechanical coefficients matrix in Table 6 quantifies these stress level dependencies.

**Figure 9:**
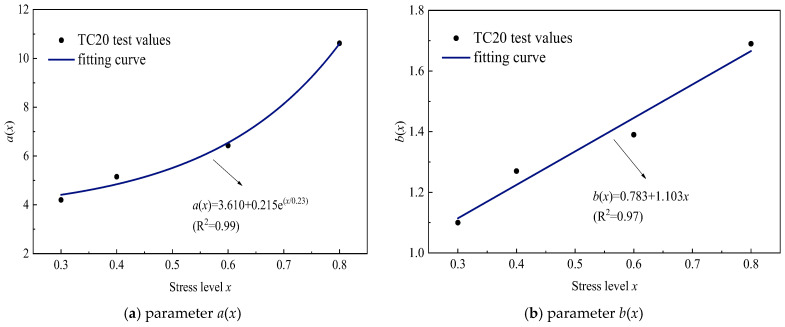
Relationship between *a*(*x*), *b*(*x*), and stress levels.

Quantitative analysis of Table 6 reveals distinct mechanical responses: The short-term relaxation coefficient *a*(*x*), representing 1 h stress relaxation, exhibits power-law dependence on normalized stress level *x* (R^2^ = 0.99), as formulated in Equation (4). Concurrently, the rate coefficient *b*(*x*) demonstrates linear proportionality with normalized stress level *x* (R^2^ = 0.97), captured by Equation (5). These stress level dependencies, graphically confirmed in Figure 9, establish that *a*(*x*) accelerates nonlinearly through stress accumulation effects, while *b*(*x*) progresses via linear viscous deformation mechanisms.

(4) $$a(x)=3.610+0.215e^{\frac{x}{0.23}}$$

(5) b(x)=0.783+1.103x

Within the established constitutive framework, the hierarchical integration of Equation (2) with experimentally determined coefficients from Equations (4) and (5) yields the predictive model Equation (6). This integrated formulation enables comprehensive simulation of stress relaxation kinetics across arbitrary normalized stress levels *x*.

(6) $$R(t)=a(\omega )(3.610+0.215e^{\frac{x}{0.23}})+b(\omega )(0.783+1.103x)\ln(t)$$

**Figure 10:**
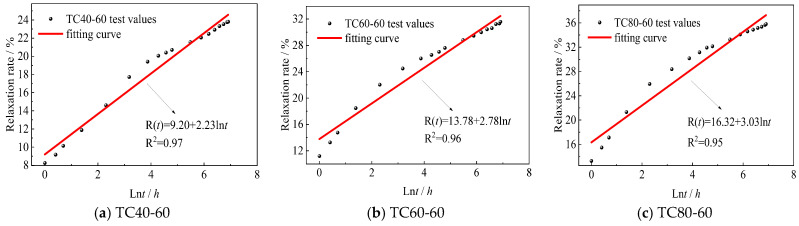
Relationship between *R*(*t*) and ln(t) at 60% stress level and different temperatures.

Then, the validated stress relaxation framework is extended to characterize thermal effects through normalized temperature *ω* = *T*/*T_g_*, where *T_g_* = 235 °C denotes the glass transition temperature (Table 1). Hierarchical implementation of Equation (1) with multi-temperature datasets (40–80 °C at stress levels *x* = 0.6) enables the determination of coefficients *a* and *b* (involving both thermal effects and stress level effects) via statistical regression analysis, as shown in Table 7. Methodological consistency with prior coefficient derivation procedures (Figure 8 and Table 6) is maintained, with Figure 10 demonstrating the preserved linear *R*(*t*)–ln(*t*) correspondence across thermally activated regimes at a 60% stress level. Similarly, at other stress levels (30%, 40%, and 80%), the determination of coefficients *a* and *b* can also be determined via statistical regression analysis, as depicted in Table 7.

**Figure 11:**
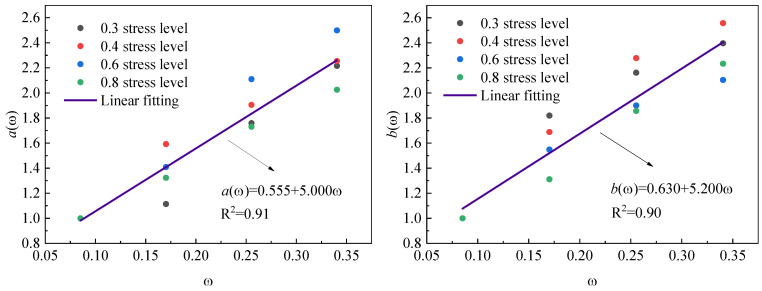
Relationship between parameters *a*(*T*), *b*(*T*), and *ω* under different stress levels.

Using the parameters in Table 7, temperature-dependent coefficients *a*(*ω*) and *b*(*ω*) are determined through normalized ratios *a*/*a*(*x*) and *b*/*b*(*x*), where stress level effects *a*(*x*) and *b*(*x*) are can be calculated using Equations (4) and (5), respectively. The calculated values of *a*(*ω*) and *b*(*ω*) in Table 8 demonstrate a positive correlation with the normalized temperature level ω. Figure 11 further reveals the linear relationships between these coefficients and ω, which can be mathematically expressed as Equations (7) and (8).

(7) $$a\left(\omega \right)=0.555+5.000\omega$$

(8) $$b\left(\omega \right)=0.630+5.200\omega$$

The integration of constitutive formulations (Equations (6)–(8)) enables the systematic characterization of stress relaxation rate *R*(*t*) evolution under coupled thermo-mechanical conditions. Through the unified formulation expressed in Equation (9), the temporal development of relaxation behavior becomes quantitatively predictable across arbitrary combinations of compressive stress levels (*x*) and normalized temperatures (*ω*).

(9) $$R(t)=(0.555+5.000\omega )(3.610+0.215e^{\frac{x}{0.23}})+(0.630+5.200\omega )(0.783+1.103x)\ln(t)$$

The predictive capabilities of Equation (9) are validated through stress relaxation projections at 1000 h and million-hour (about 114-year) timescales, as detailed in Table 9 and Table 10. Table 9 demonstrates satisfactory agreement between model predictions and experimental data across most stress levels and temperature conditions. However, deviations emerge at the 80% stress level with temperatures exceeding 40 °C, where predicted relaxation rates exceed measured values by 4–5%—a discrepancy associated with material heterogeneity and thermal control fluctuations (as evidenced by elevated variation coefficients under these conditions in Table 5). Notably, operational thresholds in practical CFRP cable anchorage systems are generally within such extreme thermo-mechanical combinations (stress level of 80% at 60 °C and 80 °C). That is to say, within the engineering parameter ranges typically encountered in practice, the prediction model maintains sufficient accuracy for structural design applications. The million-hour projections in Table 10 reveal critical temperature-dependent relaxation amplification, which underscores the necessity of incorporating temperature effects in CFRP anchorage design criteria to mitigate long-term performance deterioration under sustained thermal environments.

## 5. Conclusions

This study systematically examined the transverse compressive stress relaxation characteristics of UD-CFRP composites under varying stress levels and elevated temperature conditions. By integrating stress and thermal effects, a predictive relaxation model was established through experimental validation. The principal findings are summarized as follows.

(1) The stress relaxation behavior of UD-CFRP specimens under varying temperatures and stress levels demonstrates distinct two-stage evolution characteristics. The initial phase shows accelerated relaxation attributed to surface roughness compaction and resin matrix pore compaction. The subsequent phase manifests decelerated relaxation kinetics governed by time-dependent viscoelastic responses at the polymer matrix, especially around the fiber-resin interface, achieving quasi-equilibrium at reduced stress levels (30–40%). Notably, specimens under elevated stress conditions (60–80%) maintain progressive relaxation patterns throughout the 1000 h test duration.
(2) Elevated stress levels and temperature conditions significantly enhance both the temporal duration and magnitude of first-stage relaxation. Specifically, temperature intensification prolongs secondary relaxation completion in lower stress regimes (30–40%) while amplifying relaxation extents across all loading levels. The relaxation demonstrates marked temperature sensitivity, with thermal activation mechanisms governing the relaxation rate more profoundly than mechanical stress parameters, especially for million-hour conditions.
(3) The stress relaxation process exhibits minimal alteration of static transverse compressive strength while inducing modulus enhancement through pressure-induced porosity reduction in the resin matrix. Particularly under elevated temperatures, modulus retention demonstrates pronounced improvement (3–5% increase compared to ambient conditions) driven by thermally accelerated porosity reduction.
(4) The developed stress relaxation model demonstrates high-fidelity predictive capability across 30–80% stress levels and 20–80 °C conditions.

## Figures and Tables

**Table 1:** Material properties.

Material	Type	Tensile Strength/MPa	Elastic Modulus/GPa	Compressive Strength/MPa	*T_g_*/°C
fiber	T700 (SYT49S)	4900	240	-	-
resin	Trascite GL6831A/B	77.62	2.81	-	235

Note: *Tg* is determined by the loss modulus method.

**Table 2:** Testing stress levels and temperatures of specimens.

Specimen ID	*T*/°C	Stress Level/%	Specimen ID	*T*/°C	Stress Level/%
TC20-30	20	30	TC60-30	60	30
TC20-40		40	TC60-40		40
TC20-60		60	TC60-60		60
TC20-80		80	TC60-80		80
TC40-30	40	30	TC80-30	80	30
TC40-40		40	TC80-40		40
TC40-60		60	TC80-60		60
TC40-80		80	TC80-80		80

**Table 3:** The ending time and relaxation rate under different stages, stress levels, and temperatures.

T	Stage	Stress Level							
		30%		40%		60%		80%	
		Ending Time	Relaxation Rate	Ending Time	Relaxation Rate	Ending Time	Relaxation Rate	Ending Time	Relaxation Rate
20 °C	1st	20 h	7.85%	23 h	9.22%	25 h	17.94%	32 h	19.24%
	2nd	750 h	4.07%	825 h	4.68%	/	4.99%	/	6.87%
40 °C	1st	24 h	11.86%	27 h	15.30%	30 h	21.46%	36 h	23.96%
	2nd	780 h	5.71%	860 h	5.92%	/	6.30%	/	7.47%
60 °C	1st	27 h	16.27%	29 h	20.43%	34 h	25.21%	39 h	28.48%
	2nd	815 h	6.62%	900 h	7.30%	/	7.64%	/	8.03%
80 °C	1st	30 h	18.84%	32 h	23.41%	37 h	29.11%	42 h	37.36%
	2nd	835 h	7.22%	920 h	7.99%	/	8.21%	/	8.32%

Note: “/” means that stress relaxations had not stabilized at the testing end of 1000 h, and the relaxation rate in the second stage is taken as the value at 1000 h.

**Table 4:** Summary of relaxation rates under different temperatures and stress levels.

Stress Level	Relaxation Rate	Temperature/°C			
		20	40	60	80
0.3	Average/%	11.93	17.61	22.93	26.11
	SD/%	0.16	0.27	1.21	0.28
	CoV (%)	1.34	1.53	5.28	1.08
0.4	Average/%	13.94	21.27	27.76	31.43
	SD/%	0.17	0.50	0.21	1.70
	CoV (%)	1.22	2.35	0.77	5.41
0.6	Average/%	17.09	23.62	32.85	36.51
	SD/%	0.69	0.79	1.81	0.7
	CoV (%)	4.04	3.34	5.51	1.92
0.8	Average/%	23.30	29.73	37.32	45.68
	SD/%	0.77	0.73	0.92	0.88
	CoV (%)	3.30	2.44	2.47	1.93

**Table 5:** Post-relaxation compressive strength and modulus of different specimens.

Stress Level	Strength & Modulus	20 °C		40 °C		60 °C		80 °C	
		Strength	Modulus	Strength	Modulus	Strength	Modulus	Strength	Modulus
30%	Average/Mpa	154.08	9.68	153.95	9.89	157.48	9.84	157.85	9.80
	SD	4.40	0.29	3.45	0.54	4.36	0.42	10.80	0.56
	CoV/%	2.86	3.00	2.24	5.48	2.77	4.24	6.84	5.72
	*η*	/	1.08	/	1.11	/	1.10	/	1.10
40%	Average/%	144.10	9.92	149.80	10.20	161.42	10.14	152.68	10.33
	SD	9.09	0.49	3.04	0.69	3.47	0.15	8.14	0.80
	CoV/%	6.31	4.94	2.03	6.74	2.15	1.47	5.33	7.71
	*η*	/	1.11	/	1.14	/	1.14	/	1.16
60%	Average/%	151.36	9.81	147.70	10.27	155.76	10.07	164.94	10.41
	SD	9.78	0.75	3.00	0.78	9.39	0.47	9.00	0.77
	CoV/%	6.46	7.65	2.03	7.56	6.03	4.66	5.46	7.44
	*η*	/	1.10	/	1.15	/	1.13	/	1.17
80%	Average/%	158.13	9.79	146.88	10.04	150.94	10.05	156.90	10.30
	SD	4.58	0.36	4.07	0.51	9.51	0.40	19.85	1.02
	CoV/%	2.90	3.68	2.77	5.03	6.30	3.99	12.65	9.90
	*η*	/	1.10	/	1.13	/	1.13	/	1.15

Note: Since the compressive strength of the specimen remains largely unchanged after the relaxation test, the symbol “/” is used to denote that no comparison is made between the post-relaxation strength and the initial strength.

**Table 6:** Fitting results of *a*(*x*) and *b*(*x*) under various stress levels.

Stress Level	*a*(*x*)	*b*(*x*)	R^2^
30%	4.20	1.10	0.99
40%	5.15	1.27	0.99
60%	6.43	1.39	0.99
80%	10.62	1.69	0.98

**Table 7:** Solution results of parameters *a* and *b* under different temperatures and stress levels.

Temperature/°C	*ω*	*a*				*b*			
		Stress Level							
		0.3	0.4	0.6	0.8	0.3	0.4	0.6	0.8
20	0.085	4.40	4.83	6.53	10.58	1.11	1.22	1.44	1.67
40	0.170	4.90	7.69	9.20	14.00	2.02	2.06	2.23	2.19
60	0.255	7.74	9.20	13.78	18.32	2.40	2.78	2.70	3.10
80	0.340	9.75	10.89	16.32	21.43	2.66	3.12	3.03	3.73

Note: The values of *a* and *b* at 20 °C are calculated using Equations (4) and (5).

**Table 8:** Solution results of *a*(*ω*) and *b*(*ω*) for specimens at different stress levels and temperatures.

Temperature/°C	*ω*	*a*(*ω*)				*b*(*ω*)			
		Stress Level							
		0.3	0.4	0.6	0.8	0.3	0.4	0.6	0.8
20	0.085	1.00	1.00	1.00	1.00	1.00	1.00	1.00	1.00
40	0.170	1.11	1.59	1.41	1.32	1.82	1.69	1.55	1.31
60	0.255	1.76	1.90	2.11	1.73	2.16	2.28	1.88	1.86
80	0.340	2.22	2.25	2.50	2.03	2.40	2.56	2.10	2.23

**Table 9:** Prediction and test results of 1000 h stress relaxation rate.

Temperature/°C	Values	Stress Level			
		30%	40%	60%	80%
20	test	11.93	13.94	17.09	23.30
	prediction	12.57	13.81	17.11	22.71
40	test	17.61	21.27	23.62	29.73
	prediction	17.85	19.61	24.30	32.04
60	test	22.93	27.76	32.85	37.32
	prediction	23.13	25.63	31.50	41.70
80	test	26.11	31.43	36.51	45.68
	prediction	28.40	31.21	38.69	50.75

**Table 10:** Prediction of million-hour stress relaxation rate.

Temperature/°C	Stress Level			
	30%	40%	60%	80%
20	20.82	22.88	27.81	35.05
40	29.51	32.42	39.42	49.73
60	38.19	41.96	51.04	64.41
80	46.87	51.51	62.65	79.10

## Data Availability

The original contributions presented in this study are included in the article. Further inquiries can be directed to the corresponding author.
